# QbD-Optimized, Phospholipid-Based Elastic Nanovesicles for the Effective Delivery of 6-Gingerol: A Promising Topical Option for Pain-Related Disorders

**DOI:** 10.3390/ijms24129983

**Published:** 2023-06-10

**Authors:** Mohammed Ghazwani, Mohammed H. Alqarni, Umme Hani, Aftab Alam

**Affiliations:** 1 Department of Pharmaceutics, College of Pharmacy, King Khalid University, P.O. Box 1882, Abha 61441, Saudi Arabia; uahmed@kku.edu.sa; 2 Department of Pharmacognosy, College of Pharmacy, Prince Sattam Bin Abdulaziz University, Al Kharj 11942, Saudi Arabia; m.alqarni@psau.edu.sa (M.H.A.); a.alam@psau.edu.sa (A.A.)

**Keywords:** 6-gingerol, osteoporosis, musculoskeletal disorder, antioxidant, confocal laser scanning microscopy, transfersomes, Box–Behnken design

## Abstract

In this study, elastic nanovesicles, constructed of phospholipids optimized by Quality by Design (QbD), release 6-gingerol (6-G), a natural chemical that may alleviate osteoporosis and musculoskeletal-related pain. A 6-gingerol-loaded transfersome (6-GTF) formulation was developed using a thin film and sonication approach. 6-GTFs were optimized using BBD. Vesicle size, PDI, zeta potential, TEM, *in vitro* drug release, and antioxidant activity were evaluated for the 6-GTF formulation. The optimized 6-GTF formulation had a 160.42 nm vesicle size, a 0.259 PDI, and a −32.12 mV zeta potential. TEM showed sphericity. The 6-GTF formulation’s *in vitro* drug release was 69.21%, compared to 47.71% for the pure drug suspension. The Higuchi model best described 6-G release from transfersomes, while the Korsmeyer–Peppas model supported non-Fickian diffusion. 6-GTF had more antioxidant activity than the pure 6-G suspension. The optimized transfersome formulation was converted into a gel to improve skin retention and efficacy. The optimized gel had a spreadability of 13.46 ± 4.42 g·cm/s and an extrudability of 15.19 ± 2.01 g/cm^2^. The suspension gel had a 1.5 μg/cm^2^/h ex vivo skin penetration flux, while the 6-GTF gel had 2.71 μg/cm^2^/h. Rhodamine B-loaded TF gel reached deeper skin layers (25 μm) compared to the control solution in the CLSM study. The gel formulation’s pH, drug concentration, and texture were assessed. This study developed QbD-optimized 6-gingerol-loaded transfersomes. 6-GTF gel improved skin absorption, drug release, and antioxidant activity. These results show that the 6-GTF gel formulation has the ability to treat pain-related illnesses effectively. Hence, this study offers a possible topical treatment for conditions connected to pain.

## 1. Introduction

The global prevalence of musculoskeletal problems is estimated at millions of people of all ages [1]. Musculoskeletal disorders are the second leading cause of disability worldwide, according to Global Burden of Disease Research 2016. Painful musculoskeletal disorders afflict 20–33% of the global population [2], depending on age and diagnosis. Musculoskeletal disorders include skeletal-joint, musculotendinous, and ligamentous disorders [1,3]. This includes osteoarthritis, rheumatoid arthritis, back and neck pain, soft tissue rheumatism (tendinitis, bursitis, and myofascial pain), and sports injuries. Chronic pain hinders work, sleep, and social activities, lowering the quality of life [4]. Musculoskeletal diseases, including pathological ones, cause pain and disability, which raise healthcare costs [5]. Bone remodeling helps maintain the skeleton’s mechanical integrity. Poor diet, inactivity, and low estrogen and testosterone levels induce osteoporosis and reduced bone density [6,7]. This requires careful bone growth and resorption [8]. Natural substances are becoming more popular for lowering osteoporosis and other pain-related risks [9].

Zingiber officinale (ZO) rhizome-isolated polyphenols (gingerols) have oesteo-protective properties [10]. Much like other medicinal plants, ginger displays a wide range of therapeutic qualities owing to a variety of phytoconstituents [11,12]. Its extracts have been reported to exhibit an anti-inflammatory impact [13], a protective effect on the gastrointestinal mucosa, antioxidant effects [14], antipyretic, antibacterial, antiviral, and hypoglycemic actions [15]. Gingerols and gingerol-related compounds are two of the most active phenolic compounds in terms of their pharmacological effects [16]. The secondary metabolite 6-gingerol (6-G) is the most abundant phytochemical in ginger oleoresin [17]. In the treatment of osteoarthritis and other chronic inflammatory illnesses, 6-G has proven to be a beneficial bioactive [18]. Gingerol is a powerful antioxidant that reduces the formation of reactive oxygen species (ROS) and manages oxidative stress [19,20]. Gingerol-rich compounds reduce inflammation and pain [21]. Zerumbone, a gingerol-related polyphenol, prevents osteoclastogenesis caused by receptor activators of nuclear factor-κB (NF-κB) ligand (RANKL) [22]. This suggests ginger may help alleviate pain. Gingerols have low bioavailability due to limited water solubility and severe phase II hepatic metabolism, even though 6-gingerol is the primary bioactive component [23]. Several strategies have been used to increase the bioavailability of these compounds by various researchers [24,25]. For skin delivery of bioactive compounds, lipid-based nanocarriers such as liposomes [26], nanoemulsions [27], microemulsions [28], ethosomes [29], and solid lipid nanoparticles [30] have been frequently reported. The delivery of active chemical compounds through the barriers of the skin, namely the stratum corneum, is the most difficult aspect of developing drug delivery [31]. Transfersomes (TF), or deformable liposomes, are among the nanocarriers that are regarded as ideal for administering drugs via the skin [32]. Drug penetration is enhanced by these modified liposomes made of phospholipids and edge activators. These ultra-flexible particles can carry drugs and squeeze themselves to penetrate tissue [33]. Transfersomes improve drug penetration through the stratum corneum and deliver the drug into the epidermis [34]. Transfersomes can overcome vesicular system issues like restricted skin permeability, vesicle splitting, and drug leakage, aggregation, and fusion [35]. Transfersomes penetrate a small opening by acting as deformers and changing their shape as they go [36]. Surfactants, also known as edge activators, generate flexible liposomes and increase drug absorption through the skin. Drug concentrations increase in the deeper skin layers. These substances can be delivered by transfersomes due to their high skin penetration or poor water solubility [37].

Topical administration may avoid first-pass hepatic metabolism, increase drug duration, reduce adverse effects, and improve patient convenience [38]. Topical bioactive compounds are limited because the stratum corneum blocks the penetration of these molecules [39]. However, transfersomes, a promising lipid-based vesicular mechanism, are widely used in topical medication delivery [40].

Response Surface Methodology (RSM) studies explanatory variables that affect response variables. BBD can be used to minimize the number of experimental runs while still achieving the desired results [41]. We created 6-gingerol-loaded transfersomes (6-GTF) for osteoporosis treatment using thin-film hydration and sonication. A Box–Behnken design (BBD) optimized them. Viscosity controls medication absorption and skin residence time. Carboxy vinyl polymer (Carbopol 934) is beneficial in topical gels due to its non-appreciable rheological change in the pH range of 5–8 [42]. Vesicle shape, *in vitro* and ex vivo, CLSM, antioxidant activity, and dermatokinetic investigations were assessed for the optimized 6-gingerol-loaded transfersomes and gel formulation.

This study offers significant information on the development and optimization of transfersomes as a potential topical delivery strategy for 6-gingerol in pain-related illnesses. The clinical effectiveness and safety of these transfersomes in treating pain and enhancing patient outcomes call for further research. The study’s findings add to the body of evidence on nanocarrier-based pain management drug delivery systems, providing new directions for future study and development in this area.

## 2. Results and Discussions

### 2.1. Optimization of 6-Gingerol-Loaded Transfersomes

The Box–Behnken Design, using Design Expert^®^ software (version 13, Minneapolis, MN, USA), assessed the relationships between independent variables, i.e., phospholipid (X_1_), sodium cholate (X_2_), and sonication time (X_3_), at three levels (−1, 0, +1), and dependent responses, i.e., vesicle size (Y_1_), PDI (Y_2_), and encapsulation efficiency percentage (Y_3_). Quadratic models were found to be the best fit for both the independent and dependent responses. According to Box–Behnken Design, the compositions of various trial runs of phospholipid, sodium cholate, and sonication time are tabulated in Table 1 and Table 2. These findings confirm this study’s independent variables. ANOVA evaluated model significance. The quadratic model describes the link between independent factors and dependent responses.

### 2.2. Independent Factors’ Impact on Vesicle Size (Y_1_)

According to the experimental results, the independent variables, on vesicle size as indicated by the equation below:

vesicle size = +160.87 + 2.59 X_1_ + 3.73 X_2_ − 3.28 X_3_ + 1.45 X_1_ × 2 + 0.7125 X_1_X_3_ − 2.38 X_2_X_3_ + 9.87 X_12_ + 9.39 X_22_ + 0.0815 X_32_

**Figure 1:**
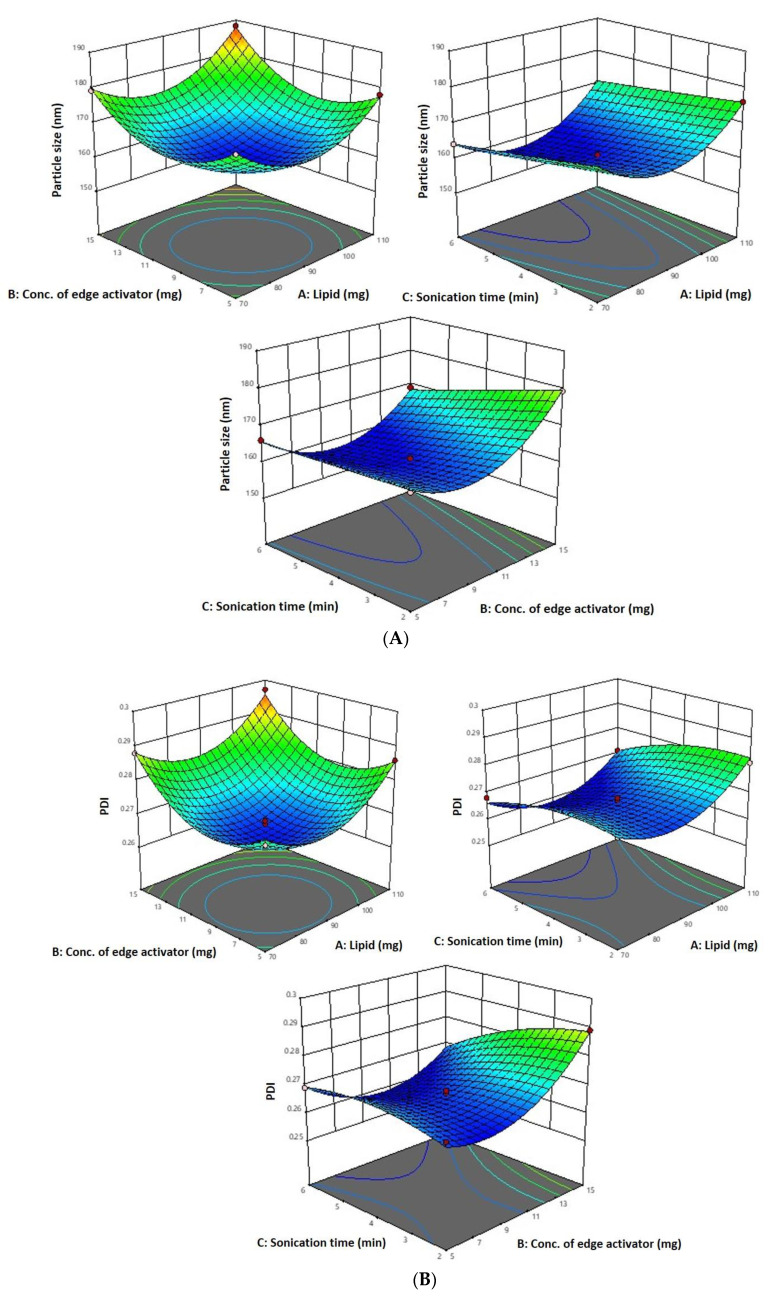

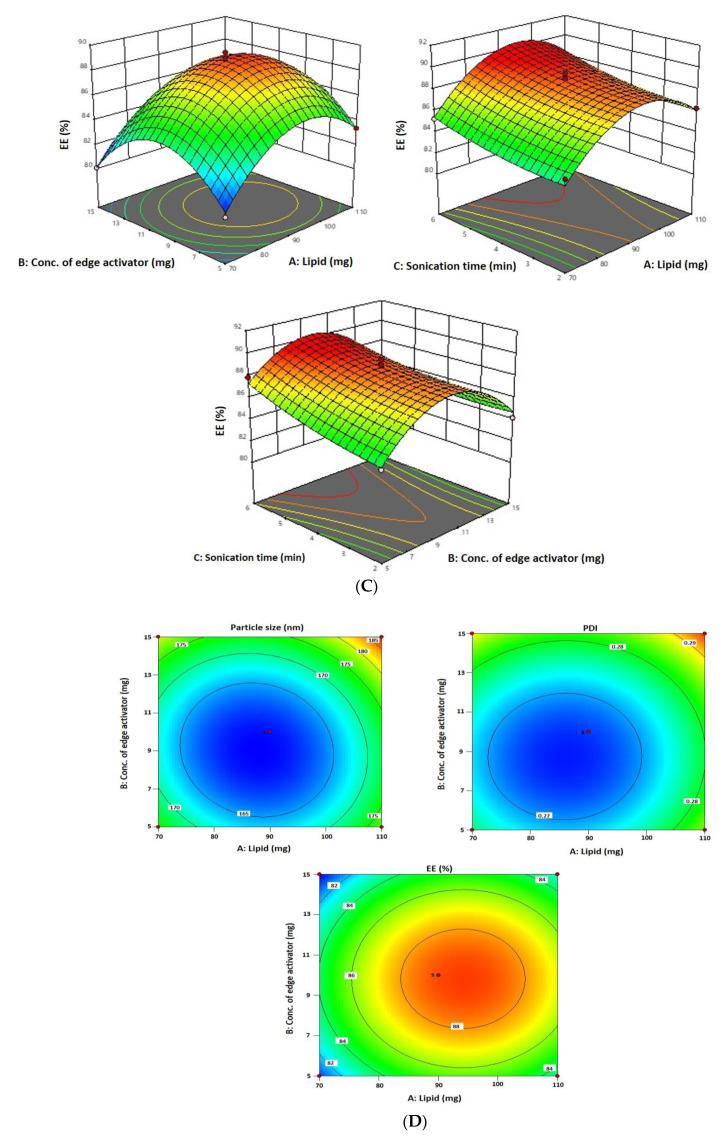
(**A**) A 3D surface plot of the effect of independent variables on vesicle size. (**B**) A 3D surface plot of the effect of independent variables on PDI. (**C**) A 3D surface plot of the effect of independent variables on entrapment efficiency. (**D**) Counterplots for response vesicle size, PDI, and entrapment efficiency of 6-GTF.

As per the above-mentioned equation and results indicated in Table 1, the 6-Gloaded transfersome vesicle sizes ranged from 160.42 nm to 188.19 nm. Figure 1A–D depict 3D and contour plots that show how influences affect size. Transfersome size marginally increases with increasing phospholipid concentration (X_1_). At high concentrations and greater availability of lipid, higher amounts of drug can be entrapped, which increases the size of the particles. To achieve the ideal vesicle size, it is crucial to have the ideal lipid content, so the minimum vesicle size is achievable by having a moderate phospholipid concentration. According to the results of our study, which are in line with earlier studies, the size of the effect increases as the phospholipid concentration in the formulation rises [43]. The vesicle size increases with the increase in sodium cholate concentration (X_2_) from 5 mg to 15 mg, as indicated in Table 1. Formulation 11 has a vesicle size of 166.08 nm (5 mg sodium cholate), formulation 7 has a vesicle size of 176.04 nm (10 mg sodium cholate), and formulation 5 has a vesicle size of 179.25 nm (15 mg sodium cholate). This is due to the edge activator’s ability to cause aggregates in excess, increasing the vesicle’s size [44]. Additionally, sonication time (X_3_) had a significant impact on vesicle size, as it reduced the size of the vesicles that may have resulted from the dispersion of agglomerated vesicles into smaller vesicles as the sonication time increased. These findings are in line with earlier studies [43,45].

### 2.3. Independent Factors’ Impact on PDI (Y_2_)

The PDI for each of the 17 runs ranged from 0.259 to 0.302, between the lower and upper limits.

PDI = +0.2602 − 0.0001 X_1_ + 0.0020 X_2_ − 0.0036 X_3_ + 0.0003 X_1_X_2_ − 0.0015 X_1_X_3_ − 0.0013 X_2_X_3_ + 0.0101 X_12_ + 0.0049 X_22_ + 0.0182 X_32_

The PDI decreases with an increase in phospholipid concentration (X_1_) from 90 mg to 110 mg and sonication time from 2 to 6 min (Figure 1B). This may be due to the vesicle size reduction caused by the sonication (X_3_), which leads to less agglomeration of vesicles by breaking them into smaller sizes with a longer sonication period, thereby increasing the homogeneity [43]. As per the above-mentioned equation, PDI increases with an increase in sodium cholate (X_2_). This is due to the edge activator’s ability to cause aggregates in excess, increasing the vesicle’s size. These findings are in line with previous reports [40].

### 2.4. Independent Factors’ Impact on Entrapment Efficiency (Y_3_)

The 6-G transfersome produced had an entrapment efficiency that varied from 80.06 to 89.43%, in accordance with the equation given below and Table 1.

Entrapment efficiency = +88.76 + 1.37 X_1_ − 0.2650 X_2_ + 1.04 X_3_ + 0.0025 X_1_X_2_ + 0.1625 X_1_X_3_ − 0.5025 X_2_X_3_ − 3.32 X_12_ − 3.73 X_22_ + 0.4762 X_32_

Since a lipophilic drug would be drawn to the lipophilic phase and be deposited there, 6-G intrinsic lipophilicity may be the reason for the steady increase in entrapment efficiency with an increase in phospholipid concentration. In the current investigation, the effectiveness of drug entrapment in formulation was found to positively correlate with the concentration of phospholipid (X^1^) and the length of the sonication (X^3^) process (Figure 1C). A high concentration of lipophilic drugs can exist in the context of high phospholipid concentrations. Because the linear increase in phospholipid concentration also influences encapsulation effectiveness, finding the ideal phospholipid concentration is crucial [43]. It was discovered that a decrease in entrapment effectiveness occurs with an increase in edge activator concentration. The encapsulation decreased as the concentration of the edge activator increased gradually. At a high concentration of edge activator, a greater amount of drug leaches out of the vesicles. With regard to the third factor, the encapsulation efficiency rises as sonication time increases, but at longer sonication times, phospholipids are degraded and vesicle structures are disrupted, leading to drug leakage and a lower EE [44].

### 2.5. Validation of Experimental Design

In order to create the ideal formulation with the lowest vesicle size, lowest PDI, and highest entrapment efficiency, BBD was used. Table 1 lists the expected values for every response and factors derived from the design. Therefore, 6-GTF formulations based on the runs, process variables, and experimental results attained using the formulations were compared to expected responses. The fact that the experimental and projected values were nearly identical supports the validity of the optimization procedure, as shown in Figure S1 (Supplementary File). The desirability values for the responses were determined to be 1, indicating the response that had the lowest prediction error and was most desired.

### 2.6. Optimized Formulation

The confirmation of the experimental design for the optimized formulation showed that the ideal formulation contained 95.16 mg of lipid, 8.91 mg of sodium cholate, and 5.94 min of sonication time. The results of the experiments were discovered to be comparable to one another, indicating the validity and dependability of the optimized formulation, as shown in Figure S1 (Supplementary File).

### 2.7. Development of Optimized 6-G-Loaded Transfersomes

The method of thin film followed by sonication was used to develop 6-G-loaded transfersomes. The detailed procedure for synthesizing 6-gingerol-loaded transfersomes can be found in the method section. After preparing 6-gingerol-loaded transfersomes, the formulation was kept in a cold, dry environment. By altering the composition and process variables, the formulation was optimized.

### 2.8. Characterization of 6-Gingerol-Loaded Transfersomes

#### 2.8.1. Vesicle Size, Zeta Potential, and Morphology

**Figure 2:**
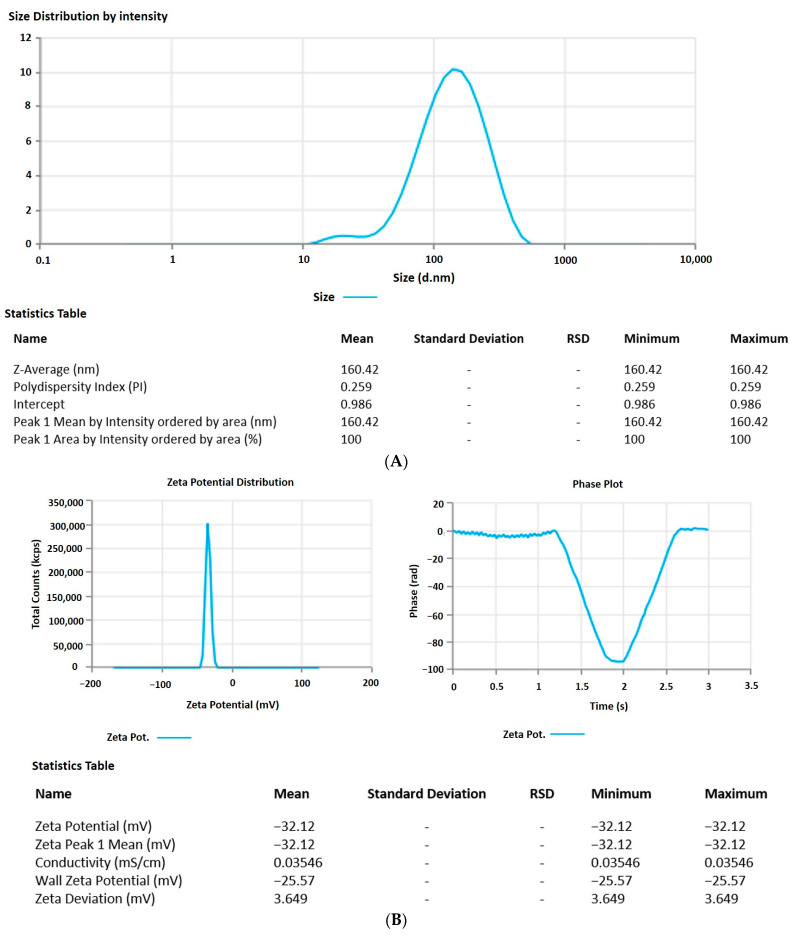

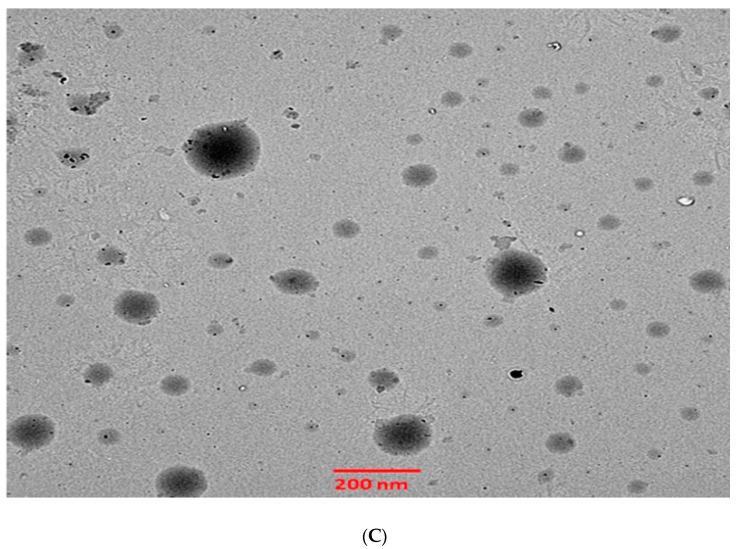
(**A**) Vesicle size of transfersomes; (**B**) Zeta potential of transfersomes; (**C**) Morphology of transfersomes.

The 6-GTF that had been prepared had an opaque look and a faintly yellowish opalescence. The PS of 6-GTF was 160.42 ± 0.25 nm on average, as shown in Figure 2A. The polydispersity index (PDI), which expresses the degree of non-uniformity in the distribution of vesicle size, is a measure of vesicle size distribution. For stability and effectiveness, nanovesicles should be monodispersed and have a PDI value of 0.3 or lower. The lower PDI value for 6-GTF in this study (0.259) denotes a monodisperse size distribution. Similar to above, the value of ZP shown in Figure 2B was frequently employed to assess the physical stability of nanoformulation during storage. The zeta potential value of the 6-GTF in this investigation was −32.12 mV, which is consistent with a stable system. Figure 2C displays the transmission electron micrographs of transfersomes. The image showed a spherical shape of vesicles without aggregation or crystal formation within the formulation [45].

#### 2.8.2. Estimation of % Entrapment Efficiency and Drug Loading

The percentage entrapment efficiency and drug loading of 6-gingerol-loaded transfersomes were found to be 89.43 ± 1.01% and 10.57%, respectively.

### 2.9. Assessment of the Transfersome Gel

#### 2.9.1. Drug Content

The most crucial aspect in the formulation of transfersomes is the drug content, and the data obtained is acceptable. It was discovered to be between 81.26 and 86.14%, demonstrating the formulation’s strong ability to hold the drug. The optimized 6-G-loaded transfersome gel formulation (1% Carbopol) contained the highest amount of drug (86.14 ± 3.14%). Dermal drug delivery techniques depend significantly on the pH of the skin, and the transfersome formulation results show that all formulations are suitable for skin delivery. The produced transfersome gels’ pH value was discovered to be between 6.98 and 7.14. The pH of the optimized 6-G-loaded transfersome gel formulation was discovered to be 7.14, as stated in Table 3.

#### 2.9.2. Spreadability and Extrudability

The most crucial characteristics for ensuring patient compliance and a uniform distribution of topical formulations are extrudability and spreadability. The extrudability and spreadability of the developed transfersome gel were discovered to range from 10.36 to 15.19 g/cm^2^ and 10.26 to 13.46 g·cm/s, respectively, as shown in Table 3. According to these results, the generated formulation spreads very well across the skin’s surface and can be easily extruded by pressing down with the thumb [46].

### 2.10. Texture Analysis

**Figure 3:**
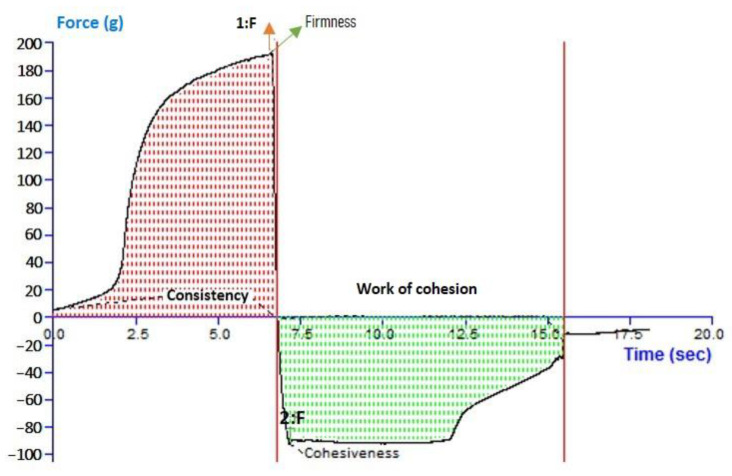
Analysis of texture of optimized transfersome gel.

For 6-GTFG, a texture analysis was conducted, and many elements like cohesion, consistency, stiffness, and index of viscosity were evaluated. The outcomes of the texture analysis of the formulation’s (6-GTFG) software output have been given, and the texture image is shown in Figure 3. The optimized 6-GTFG was found to exhibit cohesiveness (−93.55 g), consistency (783.94 g s), firmness (191.86 g), and work of cohesion (−653.11 g s), respectively.

#### 2.10.1. *In Vitro* Drug Release 

**Figure 4:**
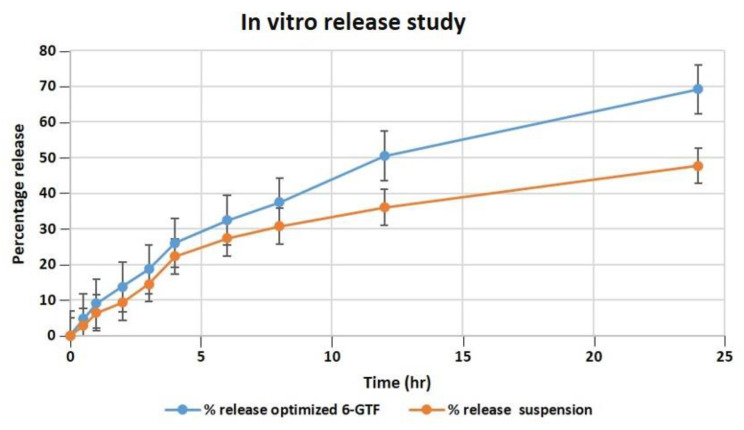
Comparative *in vitro* release profile of 6-GTF through a dialysis membrane.

Our studies found that after 24 h, the optimized transfersomes released 69.21 percent of the drug, while the pure drug suspension released 47.71 percent, as shown in Figure 4. The *in vitro* data revealed a biphasic release process, with a quick release from the surface-adsorbed drug in the first 2 h, followed by a gradual release profile for up to 24 h [47]. The Korsmeyer–Peppas model exhibited the highest R^2^ value of 0.9901 and an *n* value of 0.697, indicating the pattern of non-Fickian diffusion release of drug, and it follows the Higuchi model release pattern with an R^2^ value of 0.995, followed by zero- and first-order R^2^ values of 0.9291 and 0.9893, respectively. According to the data of the *in vitro* drug release investigation, the optimized TF formulation has a suitable structure capable of entrapping 6-G to ensure their gradual release as the drug was successfully entrapped by the transfersomes, enabling a sustained release that could be effective in a drug delivery system that demands prolonged release profiles.

#### 2.10.2. Determination of Antioxidant activity 

Using the ABTS assay, the antioxidant capability of 6-gingerol and its developed formulation was assessed. The ability of 6-gingerol to operate as reducing agents is based on their redox capabilities; this ability is typically linked to the presence of reductants, which break the free radical chain by donating a hydrogen atom or prevent the development of peroxide to produce antioxidant action [48]. With experiments proving the drug’s effectiveness in scavenging free radicals and reducing lipid peroxidation, 6-gingerol has been found to have considerable antioxidant potential. Both the pure 6-G and the optimized 6-GTF formulations had their antioxidant properties evaluated and contrasted with a standard solution. Antioxidant activity ranged from 91.14 ± 0.24 percent in a standard solution, 67.01 ± 1.11 percent in free 6-G, and 76.89 ± 3.19 percent in a 6-GTF formulation [49].

#### 2.10.3. Ex Vivo Study of the Permeation of Skin

**Figure 5:**
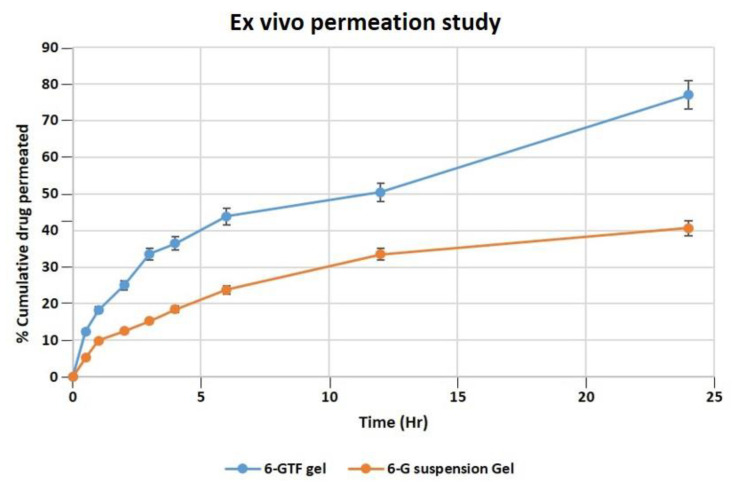
Permeation of 6-G from 6-GTF gel and 6-G suspension gel over the rat excised skin in a unit of time.

According to an ex vivo skin permeation investigation, 40.63% of 6-G from 6-G suspension gel permeated the skin. While the optimized 6-GTF gel formulation demonstrated 77.01% 6-G permeation across rat skin, as demonstrated in Figure 5. The 6-GTF gel showed initial burst release (36.45%), which is higher than that of the 6-G suspension gel (18.45) after 4 h. The drug transport profile from the 6-GTFG formulation across rat skin was found to have a considerably higher flux value, i.e., 2.71 μg/cm^2^/h compared to the 6-G suspension gel, which showed a flux of 1.5 μg/cm^2^/h. The considerable distinction in the percentage transport of drugs might be due to the incorporation of 6-G into the optimized TF gel system [46], which could have been due to the nanosized TF vesicles. However, due to transfersomes’ flexibility, they are able to penetrate the skin and overcome the barrier function by squeezing themselves through the pores of the stratum corneum.

#### 2.10.4. CLSM

Confocal microscopic images showed that the rhodamine B hydroalcoholic solution only contacted the topmost layers of skin and that the dye’s fluorescence permeated as deep as 5.0 µm (Figure S2B; Supplementary File). Rhodamine B-loaded TF gels were penetrated 25.0 µm into the skin (Figure S2A; Supplementary File). The finding of higher fluorescence intensity indicates that the formulation has a sustained presence and greater levels in the deeper layers of the skin. Using the newly developed TF formulation, rhodamine B was successfully delivered into the dermal layers of rat skin.

#### 2.10.5. Dermatokinetics for Optimized Formulation

**Figure 6:**
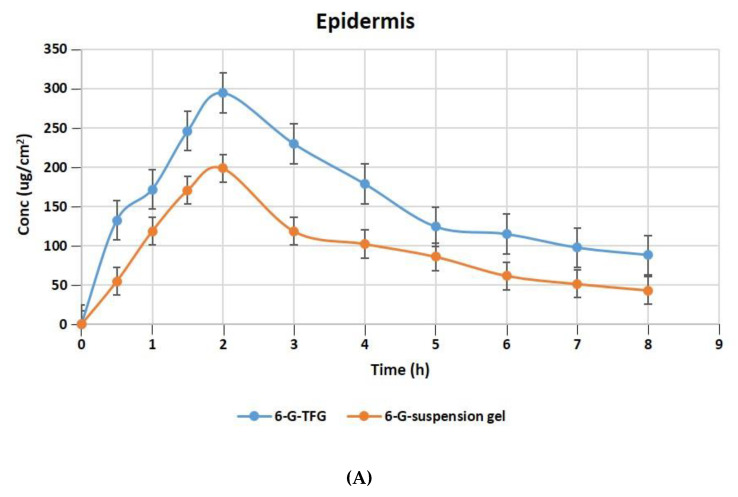

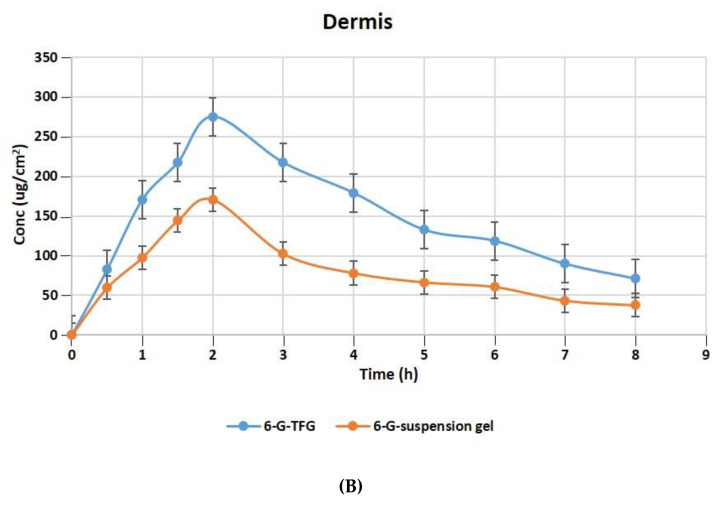
6-G concentration on rat skin (**A**) epidermis (**B**) dermis after application to the skin of 6-GTF gel and 6-G-suspension gel.

The relative concentration of 6-G in the layers of rat skin’s dermis and epidermis following treatment with gel formulations of 6-GTFG and 6-G-suspension gel at different time intervals is displayed in Figure 6A,B. The dermatokinetic parameter values are presented in Table 4. The excised rat skin treated with 6-GTF gel and 6-G-suspension gel revealed that the 6-GTF gel produced much larger concentrations of 6-G in the epidermis, with values of Cskin max of 294.61 ± 2.14 μg/cm^2^ and 198.49 ± 1.11 μg/cm^2^, respectively, and in the dermis, the values of Cskin max for 6-GTFG and 6-G suspension gel were found to be 274.92 ± 1.49 μg/cm^2^ and 170.70 ± 0.99 μg/cm^2^, respectively. The 6-GTFG delivery demonstrated quick drug absorption when compared to the suspension gel formulation, reaching the Cmax in the dermis and epidermis after topical administration within 1.5 and 2 h, respectively. The AUC_0–8h_ of 6-G in 6-GTFG was much higher than the 6-G suspension gel in the epidermis, and it was found to be 1286.16 ± 2.49 μg/cm^2^ h and 762.99 ± 0.99 μg/cm^2^ h, respectively. In dermis, the AUC_0-8_ of 6-G concentration in 6-GTFG was considerably greater when compared to 6-G suspension gel; it was found to be 1213.16 ± 1.01 μg/cm^2^ h and 648.34 ± 0.11 μg/cm^2^ h, respectively. Vesicles in 6-GTF gel improve drug absorption and retention in skin layers. It was discovered that the 6-G concentration fell over the course of the experiment until 8 h had passed and the drug’s concentration was detectable. The above results of the TF delivery suggest increased penetration of the delivery tool when applied topically, which may be related to the earlier findings of the confocal laser microscopic study and skin permeation study, where the increased penetration of the formulation was obvious due to its elasticity and the presence of an edge activator.

### 2.11. Stability Studies

Physiochemical stability tests were performed to determine the period of time the 6-GTF gel may be expected to remain stable after it has been prepared. Based on the findings, 6-GTF gel is the best option. The product met all required specifications for color, clarity, pH, and other parameters shown in Table 5.

## 3. Materials and Method

### 3.1. Material

6-Gingerol was procured from Sigma Aldrich (Mumbai, India); sodium cholate and methanol were obtained from Merck Pvt. Ltd. (Mumbai, India). Phospholipid, Carbopol 934, and triethanolamine were purchased from SD Fine Chemicals Ltd. (Mumbai, India). Dialysis membrane bags were procured from Sigma-Aldrich Co., St. Louis, MO, USA. All other substances were of an experimental grade, such as sodium dihydrogen phosphate and sodium chloride, which were provided by S D Fine Chemicals Limited, Mumbai, India.

### 3.2. Methods

#### 3.2.1. Developing Transfersomes Loaded with 6-Gingerol (6-GTF)

The 6-GTF were prepared by the traditional method of thin film hydration followed by sonication. Initially, to produce vesicle suspension, a weighed amount of phospholipid, drug (6-G), and edge activator (sodium cholate) was dissolved in a chloroform: methanol (5:1 *v*/*v*) mixture. The dry lipid film was prepared by evaporating the solvent in the rotary evaporator. The lipid layer was vacuum-dried to remove the solvents. The thin dried layer was hydrated with distilled water (10 mL), and it was then allowed to rotate for 1 h at 90 °C at 60 rpm to swell. Further, the formulation was sonicated using a probe sonicator (UP100H, Hielscher GmbH, Teltow, Germany) for 5.94 min, and then extruded through membrane filters (0.25 µm). After the preparation, the formulation was stored in a cool, dry place. The formulation was optimized by changing the formulation and process variables [50].

#### 3.2.2. Transfersome Optimization Using Box–Behnken Design (BBD)

Using response surface methodology (RSM), a three-factor, three-level Box–Behnken design (BBD) was utilized to optimize the transfersomes. Many researchers found that phospholipid (X_1_), edge activator (X_2_), and sonication time (X_3_) affect formulation stability and entrapment efficiency; hence, they were chosen as independent variables. Vesicle size (Y_1_), polydispersity index (PDI) (Y_2_), and entrapment efficiency (Y_3_) were chosen as dependent variables for optimization. After many preliminary experiments, phospholipid, sodium cholate, and probe sonication time were selected in the ranges of 70–110 mg, 5–15 mg, and 2–6 min, respectively, as independent variables for 6-gingerol-loaded transfersome optimization (Table 6). Table 1 shows 17 BBD-prepared trial formulations of various factor combinations, including five center points. The information was put into BBD, and mathematical modeling was used to analyze the results. ANOVA, in addition to the coefficient of correlation (R_2_), adjusted R_2_, anticipated R_2_, and predicted residual sum of squares, was used to analyze the data fitting the selected quadratic second-order model. The optimum conditions for the development of transfersomes were determined with the use of the numerical desirability function and the graphical optimization technique. The study maintained all other formulation and processing characteristics as constants.

#### 3.2.3. Measurement of Vesicle Size, PDI and Zeta Potential for 6-GTF

Using dynamic light scattering, the vesicles’ size, size distribution curve, and electric potential were determined using a Malvern Zetasizer, ver.7.11, serial number MAL1121994 (Malvern Panalytical Ltd., Malvern, United Kingdom). Prior to the study of the aforementioned parameters, the optimized 6-GTF was diluted roughly 100 times. The temperature was maintained at about 25 ± 2 °C, while the scattering angle was kept at 90° [51]. The same procedure was followed for the blank formulation.

#### 3.2.4. Determination of 6-G Encapsulation Efficiency (%EE) and % Drug Loading (DL)

The ultracentrifugation technique (Beckman Coulter India Pvt. Ltd., New Delhi, India) was used to determine % EE of 6-G-loaded in the transferosomes. The obtained formulations were centrifuged for one hour at 6000 rpm. The supernatant was withdrawn, diluted, and filtered through a membrane filter (0.25 μm), and the drug concentration was determined at 278 nm using a UV spectrophotometer (Shimadzu, Tokyo, Japan). Drug entrapment effectiveness is an important consideration when assessing transfersomes ability to load drugs. This metric is influenced by the drug’s physicochemical characteristics, development process, and formulation considerations. The blank sample was prepared using the same method. The drug was not incorporated into the formulation’s development. The following equation was used to determine the percentage of the drug encapsulated and loaded [35,52].

$$Encapuslation\;efficiency\;\left(\%\right)=\frac{\left(Total\;drug-Free\;drug\right)}{Total\;drug}\times 100$$

$$Drug\;loading\;\left(\%\right)=\frac{Amount\;of\;drug\;entraped}{Total\;amount\;of\;drug\;and\;lipid}\times 100$$

#### 3.2.5. Morphological Study

Transmission electron microscopy (TEM) was used to study the surface morphology of the optimized transfersomes utilizing a microscope (FEI Company, Eindhoven, Netherlands). A diluted drop of TF was applied to a copper grid that had been covered with carbon. The sample was dyed with 1% phosphotungstic acid for 10 s, and then it was set to air-dry at room temperature before being examined by a TEM at 70 kV [53]. 

#### 3.2.6. Preparation of 6-Gingerol-Based TF Gel (6-GTFG)

The optimized 6-G transfersomes (6-GTF) were incorporated into a gel (6-GTFG). Carbopol 934 was employed as the gelling agent. In order to create the dispersion, distilled water was first gently added to the Carbopol 934. After that, the mixture was allowed to sit overnight in the dark to fully swell. Triethanolamine (0.5% *w*/*v*) was gradually added to the dispersion to further neutralize it, enabling the development of a clear, viscous gel. The Carbopol gels at 0.5%, 1%, and 2% were made. Finally, the optimized 6-GTF formulation was gently added to the gel while being continuously stirred [54].

#### 3.2.7. Evaluation of 6-G Based TF Gel (6-GTFG)

##### Organoleptic Assessment and pH Determination

The resulting formulations’ appearance or homogeneity were visually inspected for any grittiness or agglomerates. Additionally, a precise weight of the 6-GTF gel formulation (1 g) was used to dilute it with double-distilled water. The pH value of the developed formulation was evaluated at room temperature using a pH meter (Mettler Toledo, Ohio, United States).

##### Drug Content

A weighed amount (100 mg) of the produced gel was dispersed in phosphate-buffered saline (PBS) (pH 7.4) (100 mL). To ensure complete drug solubility, the volumetric flask holding the gel solution was agitated on a mechanical shaker for 2 h. After this, the solution was filtered using a membrane (pore size 0.45 mm). Using a UV–visible spectrophotometer and solvent as a blank (followed by the same procedure without the incorporation of drug in a gel formulation), the absorbance of the sample was calculated at 278 nm [55].

##### Spreadability and Extrudability Determination

The spreadability and extrudability of the developed formulation are critical factors in determining uniform distribution and spreading. The spreadability of the formulations describes the area where they can easily spread following skin application. A reference load (500 g) was administered to the top slide for five minutes while the experimental formulations (1 g) were sandwiched in between two glass slides [56]. The time (in seconds) required to separate the two slides served as a gauge of their spreadability. The formula used to calculate the spreadability, as follows:

S=M· L/T

*S* stands for spreadability, *M* for the weight supported by the higher slide, *L* for the glass slide’s length, and *T* for the processing time.

Extrudability is defined as the quantity of material in g required to extrude no less than a half-centimeter ribbon of the formulation from a breakable tube within ten seconds. The 6-G-loaded transfersome gel (20 g) was packed in a collapsible tube, and the crimped end of the tube was pushed to force the gel out when the cap was removed [57]. The following equation was used to determine the extrudability (g/cm^2^):

$$Extrudability=\frac{Applied\;weight\;to\;the\;tube\;\left(gm\right)}{Area\;\left(cm^{2}\right)}\;$$

##### Gel Texture Evaluation

The texture of the generated 6-GTFG was inspected using a texture analyzer (TA.XT Plus Texture Analyzer, Stable Micro Systems Ltd., Surrey, UK). A 100 mL glass beaker was filled with 100 g of the optimized gel formulation (6-GTFG), and the surface was maintained as smooth as possible to avoid air bubble entrapment. The analytical probe was inserted twice into the gel-filled beaker at a depth of 15 mm, moving slowly (2 mm per s) and allowing 20 s between compressions. A force curve that includes mechanical traits like cohesiveness, consistency, cohesiveness index, and hardness was used to describe the results [58].

##### *In Vitro* 6-G Release and Release Kinetic Modeling

- Cellophane membrane activation

To remove glycerin and sulfur compounds and to open pores, the dialysis membrane is always activated prior to *in vitro* experiments. By washing the dialysis membrane under running water for three to four hours, the glycerin was removed. Using a 0.3% *w/v* sodium sulfide solution at 80 °C for 1 min, the sulfur compounds were removed. The tube was first rinsed in hot water (60 °C) for 2 min, then acidified with sulfuric acid (0.2% *v*/*v*), and finally treated with hot water to remove the acid. Membranes preserve the majority of proteins with molecular weights of 12,000 or more after this treatment [59].

- Conducting *in vitro* release studies

The *in vitro* drug release from the optimized 6-GTF formulation under standard circumstances was examined using the cellophane dialysis membrane bag technique (molecular weight cutoff 12,400 Da, Sigma-Aldrich Co., St. Louis, MO, USA). In the dialysis bags, 2 mL of the 6-GTF formulation and 6-G suspension (10 mg of drug was dissolved in methanol and made up to 10 mL with a suitable solvent) were loaded before being bound at both ends. The dialysis bags were submerged in a beaker filled with 25 mL of PBS (pH 7.4) that was magnetically stirred at 100 rpm and 37 ± 0.2 °C throughout the experiment. The samples were taken at various intervals (0, 0.5, 1, 2, 4, 6, 8, 12, and 24 h) from the beaker while sink conditions were maintained, and they were UV spectrophotometrically examined for the drug content against the PBS pH 7.4 as a blank [60].

Each measurement was made in triplicate. By fitting the results into various models, including zero-order, first-order, Korsmeyer–Peppas, and Higuchi mathematical models, the drug release mechanism was further evaluated. In accordance with the great value of the coefficient of correlation (R2), the model that best describes the 6-G release order was chosen [58].

$$\begin{array}{l} \%\;Drug\;release\\ =\frac{Conc.\;\left(µg/mL\right)\;\times \;DF\;\times \;Volume\;of\;the\;release\;medium\;\left(mL\right)}{Initial\;dose\;\left(µg\right)}\times 100 \end{array}$$

where *DF* stands for dilution factor.

##### Ex Vivo Skin Permeation Study

- Preparation of skin

The skins of healthy Wistar rats were removed for the ex vivo skin permeation study and dermatokinetic investigation. The skin permeation experiments and dermatokinetics on rodent skin were properly approved by the Standing Committee of Bioethics Research at Prince Sattam bin Abdulaziz University in Al-Kharj, Saudi Arabia (SCBR-020-2023). The 1 cm^2^ of abdomen skin of the rat was cut, and the hair was removed by the use of hair removal cream. Manual removal of the subcutaneous tissue was performed, and any remaining clinging fat was removed by wiping the dermis side with isopropyl alcohol. The skin’s entire thickness was cleaned with PBS 7.4. Later, the skin was covered in aluminum foil and kept at −20 °C until use (used within 2 weeks of preparation) [61]. In the Franz diffusion cell assembly, the skin was placed in such a way that the stratum corneum side faces the donor compartment and the dermis side faces the receiver compartment.

#### 3.2.8. Permeation Study

“Franz diffusion cells”, having a diffusion surface area of 0.785 cm^2^ over rat skin, were used to conduct a skin permeation experiment. In the donor compartment, 1 gm of the 6-G-loaded transfersome gel formulation was placed. Then, 10 mL of phosphate-buffered saline (pH 7.4) was placed in the receiver cell, which was maintained at 37 ± 1 °C during the experiment and stirred with a magnetic bead rotating at 600 rpm [62]. Aliquots (1 mL) of samples were taken out and replaced with the dissolving media at predetermined intervals of time, as performed in *in vitro* studies. The withdrawn samples underwent additional filtering, were diluted with PBS 7.4, and were measured spectrophotometrically using UV spectrophotometry for their drug content by taking PBS as a blank [63]. The slope of the linear component of the plot was used as the steady-state flux (*Jss*) to calculate the cumulative amount of 6-G that penetrates per unit area over time. The following equation was used to measure the permeability coefficient (*Kp*):

$$Kp=\frac{Jss}{Cv}\;\;$$

where *Cv* is the formulation’s overall donor concentration [64].

#### 3.2.9. Confocal Laser Scanning Microscopy (CLSM)

The CLSM (Leica Microsystems GmbH, Wetzlar, Germany) investigation measured the penetration depth of the produced TF vesicles. Rhodamine B dye was added to the optimized TF gel formulation, and CLSM measurements of the rat skin’s penetration depth were made in comparison to the control (solution of rhodamine B-hydroethanolic). Franz diffusion cells were used to conduct a study of skin permeation for 8 h using rhodamine B-loaded TF gel and a hydroethanolic solution of rhodamine B for the CLSM experiment. Glass slides of skin samples were made and viewed under a CLMS microscope, and probe dye penetration depth was measured [65].

#### 3.2.10. 2,2′-Azino-bis(3-Ethylbenzothiazoline-6-sulphonic) Acid (ABTS) Radical Scavenging Activity

The ability of the nanocarriers to scavenge the free radical ABTS was used to measure their antioxidant activity. To create an ABTS free radical, a stock solution of 7 mM ABTS was combined with 2.45 mM potassium persulfate and agitated in the dark for 12 h. The solution was diluted with ethanol prior to use until it had an absorbance of 0.7. Then, a standard solution (ascorbic acid) was mixed with the ABTS+ solution and incubated for 10 min in a 37 °C water bath. After that, various concentrations (10–100 µg/mL) of all samples were prepared in ethanol, 3 mL of the solution and 1 mL of the samples were combined, and the absorbance was measured at 734 nm using a UV spectrophotometer after 6 min [66]. Finally, the following equation was used to compute ABTS scavenging activity:

$$Scavenging\;\left(\%\right)=\frac{\left(Absorbance\;blank-Absorbance\;sample\right)}{Absorbance\;blank}\times 100$$

#### 3.2.11. Dermatokinetics

Wistar rats’ skins that had been removed were employed in the study. As mentioned in the section on ex vivo skin permeation, the skin tissue was prepared. For the experiments, Franz cell diffusion assembly (PermeGear, Inc., Hellertown, PA, USA) was used, as discussed under permeation studies. In this instance, the entire skin was peeled off of the Franz cell at the appropriate sampling intervals. In order to separate the epidermis from the dermis, the skin was cleansed three times to eliminate any remaining adhesive formulation. For complete drug extraction, each of the sections was cut into small pieces and macerated in methanol (5 mL) for 24 h. The filtrate was examined using the UV method after the solution was filtered through a membrane (0.45 µm). The concentration of 6-gingerol per square centimeter of skin versus time was plotted separately for the epidermis and dermis. The dermatokinetic parameters such as *Tskin max*, *Cskin max*, AUC_0–8h_, and *Ke* were also calculated [67]. An equation was used to fit the collected data into a single compartment open model.

$$Cskin=\frac{Kp\cdot \;Cskin\;max}{Kp-Ke}\;\left(e^{-Kpt}-e^{-Ket}\right)$$

where *Cskin* is the drug’s concentration in the skin at time *t*, *Kp* is the constant for dermal penetration, *Cmax* skin is the highest concentration that can be found in the skin, and *Ke* is the constant for skin elimination.

#### 3.2.12. Stability Studies

Short-term accelerated stability studies were conducted according to ICH Q1 A (R2) requirements, whereby the 6-GTF gel was subjected to testing for a total of six months. In order to test the stability of the optimized 6-GTF gel, it was kept at a temperature of 25 ± 2 °C and a relative humidity of 60 ± 5% RH in an aluminum-covered container. Throughout the process of developing and using the gel, measurements were made of its appearance, clarity, homogeneity, pH, spreadability, extrudability, and 6-G content [58].

## 4. Conclusions

The thin film approach followed by sonication was utilized to develop the 6-gingerol-loaded transfersomes. Quality-by-Design (QbD) was used to optimize 6-gingerol-loaded transfersomes. The 6-GTF gel formulation showed improved skin absorption, sustained drug release, and increased antioxidant activity. These advances lay the groundwork for a future topical treatment for pain linked to osteoporosis and musculoskeletal diseases, giving tailored relief and improving patient outcomes. To verify the effectiveness and safety of this formulation, future research should concentrate on clinical studies and additional optimization. If it is successful, this strategy could offer a useful substitute for pain management, bringing more effective and tailored relief to people with these illnesses.

## Figures and Tables

**Table 1:** Formulation runs and responses of various compositions as per BBD.

Formulations	Independent Variables			Dependent Variables		
	Phospholipid (mg) (X_1_)	Sodium Cholate (mg) (X_2_)	Sonication Time (min) (X_3_)	Vesicle Size (nm) (Y_1_)	PDI (Y_2_)	Entrapment Efficiency (%) (Y_3_)
1	70	10	2	173.15	0.272	84.22
2	90	15	6	169.05	0.267	85.97
3	90	5	2	166.87	0.269	84.04
4	90	10	4	161.05	0.268	88.67
5	70	15	4	179.25	0.288	80.06
6	110	5	4	178.11	0.286	83.36
7	110	10	2	176.04	0.281	86.25
8	70	5	4	174.98	0.276	80.25
9	70	10	6	164.18	0.268	85.26
10	90	10	4	160.97	0.266	88.98
11	90	5	6	166.08	0.269	87.85
12	90	10	4	160.42	0.259	89.43
13	110	10	6	169.92	0.271	87.94
14	90	15	2	179.38	0.289	84.17
15	90	10	4	160.88	0.267	88.48
16	90	10	4	161.04	0.268	88.24
17	110	15	4	188.19	0.297	83.18

X_1_ = Phospholipid (mg), X_2_ = Sodium cholate (mg), X_3_ = Sonication time (min), Y_1_ = Vesicle size (nm), Y_2_ = PDI, and Y_3_ = EE (%).

**Table 2:** Results of regression analysis of Y_1_, Y_2_, and Y_3_ responses.

Quadratic Model	R^2^	Adjusted R^2^	Predicted R^2^	SD	%CV	*p*-Values		
						X_1_	X_2_	X_3_
Response (Y_1_)	0.9965	0.9921	0.9480	0.7448	0.4382	<0.0001	<0.0001	<0.0001
Response (Y_2_)	0.9845	0.9647	0.8158	0.0019	0.6752	0.0121	0.0030	0.0059
Response (Y_3_)	0.9825	0.9601	0.8097	0.5877	0.6860	<0.0001	0.003	0.0019

**Table 3:** Results of 6-G-loaded transfersome gel formulations.

Formulation Gel Code with Carbopol Conc.	Homogeneity	pH	Drug Content (%)	Spreadability (g·cm/s)	Extrudability (g/cm^2^)
TF1 (0.5%)	Homogeneous	6.98	81.26 ± 2.14	10.26 ± 1.29	10.36 ± 1.11
TF2 (1%)	Homogeneous	7.14	86.14 ± 3.14	13.46 ± 4.42	15.19 ± 2.01
TF3 (2%)	Homogeneous	7.09	85.14 ± 2.30	11.12 ± 2.64	13.18 ± 0.78

**Table 4:** Dermatokinetic parameters (Mean ± SD) of 6-GTF-gel and 6-G suspension-gel.

Dermatokinetics Parameters	6-GTF-Gel		6-G Suspension Gel	
	Epidermis Mean ± SD	Dermis Mean ± SD	Epidermis Mean ± SD	Dermis Mean ± SD
T_skin max_ (h)	2	2	2	2
C_skin max_ (μg/cm^2^)	294.61 ± 2.14	274.92 ± 1.49	198.49 ± 1.11	170.70 ± 0.99
AUC_0–8_ (μg/cm^2^ h)	1286.16 ± 2.49	1213.16 ± 1.01	762.99 ± 0.99	648.34 ± 0.11
Ke (h^−1^)	0.113 ± 0.14	0.113 ± 0.88	0.128 ± 0.46	0.136 ± 0.89

Tskin max = Time to maximum concentration, Cskin max = Maximum concentration, AUC = Area under curve, Ke = Elimination rate constant.

**Table 5:** Stability studies for optimized 6-GTF gel stored at 25 ± 2 °C/60 ± 5% and 4 ± 2 °C.

Evaluation Parameters	Initial	1 Month		3 Months		6 Months	
		4 ± 2 °C	25 ± 2 °C/60 ± 5% RH	4 ± 2 °C	25 ± 2 °C/60 ± 5% RH	4 ± 2 °C	25 ± 2 °C/60 ± 5% RH
Color	Slightly yellowish	Slightly yellowish	Slightly yellowish	Slightly yellowish	Slightly yellowish	Slightly yellowish	Slightly yellowish
Appearance	Translucent	Translucent	Translucent	Translucent	Translucent	Translucent	Translucent
Homogeneity	***	***	***	***	**	***	**
Clarity	√	√	√	√	√	√	√
pH	7.14	7.14	7.09	7.07	7.04	7.06	7.03
Washability	Washable	Washable	Washable	Washable	Washable	Washable	Washable

** Good, *** Excellent, √ Yes.

**Table 6:** Independent and dependent variables used in BBD for the development of 6-GTF.

Variables	Used Levels		
	Low (−1)	Medium (0)	High (+1)
Independent variables			
X_1_ = Phospholipid (mg)	70	90	110
X_2_ = Sodium cholate (mg)	5	10	15
X_3_ = Sonication time (min)	2	4	6
Dependent variables			
Y_1_ = Vesicle size (nm)		Minimize	
Y_2_ = PDI		Minimize	
Y_3_ = EE (%)		Maximize	

## Data Availability

Not applicable.

## Supplementary Materials

The supporting information can be downloaded at: https://www.mdpi.com/article/10.3390/ijms24129983/s1.
