# Supplementary material for: QbD-Optimized, Phospholipid-Based Elastic Nanovesicles for the Effective Delivery of 6-Gingerol: A Promising Topical Option for Pain-Related Disorders
Source: Int J Mol Sci. 2023 Jun 10;24(12):9983. doi: 10.3390/ijms24129983 (PMC10298657; doi:10.3390/ijms24129983)
Supplement: Supplementary file 1 [file ijms-24-09983-s001.zip › ijms-2422228-supplementary.pdf]

Supplementary Figure S1

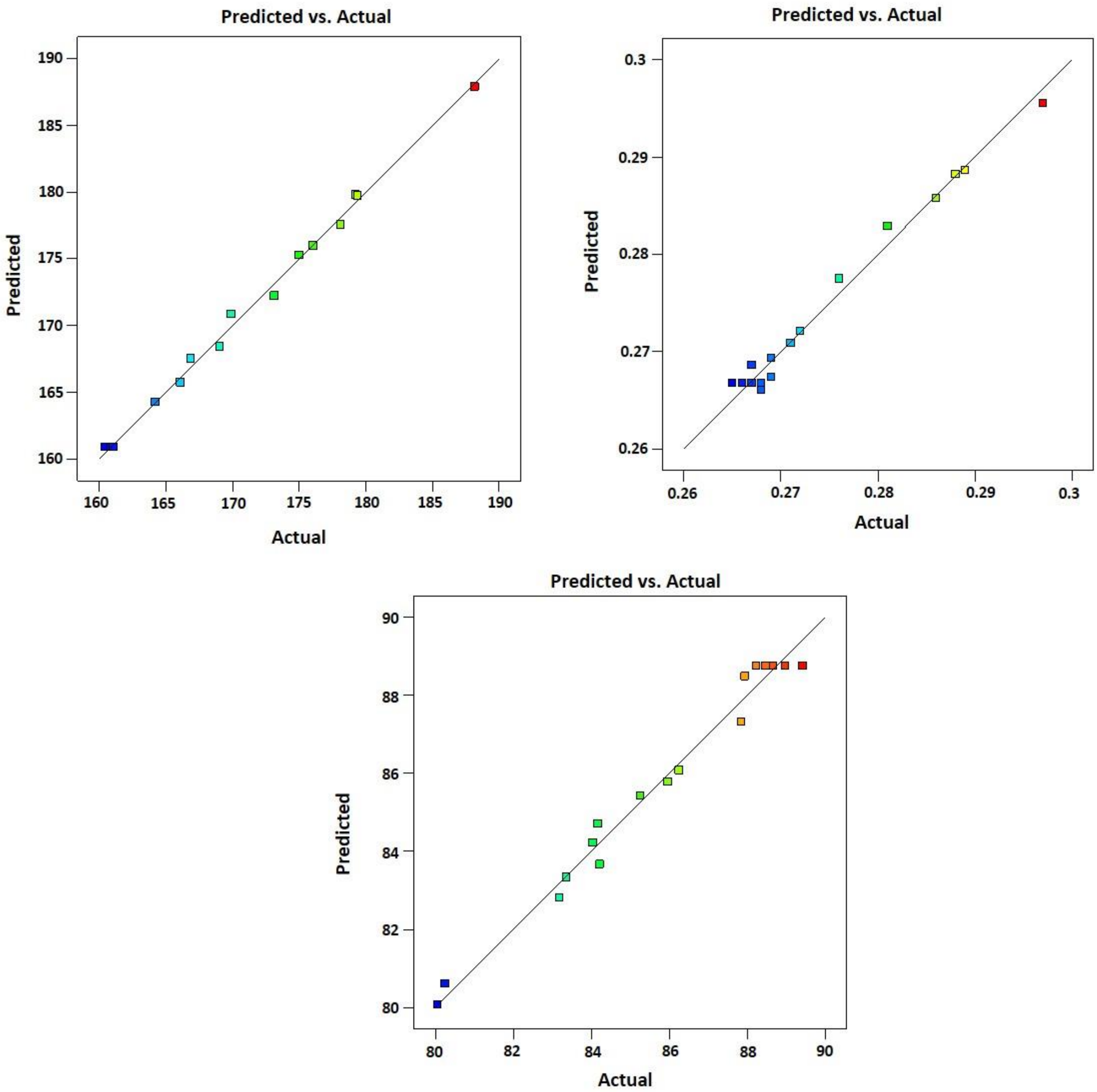

Figure S1. The predicted vs Actual plot for various responses

Supplementary Figure S2

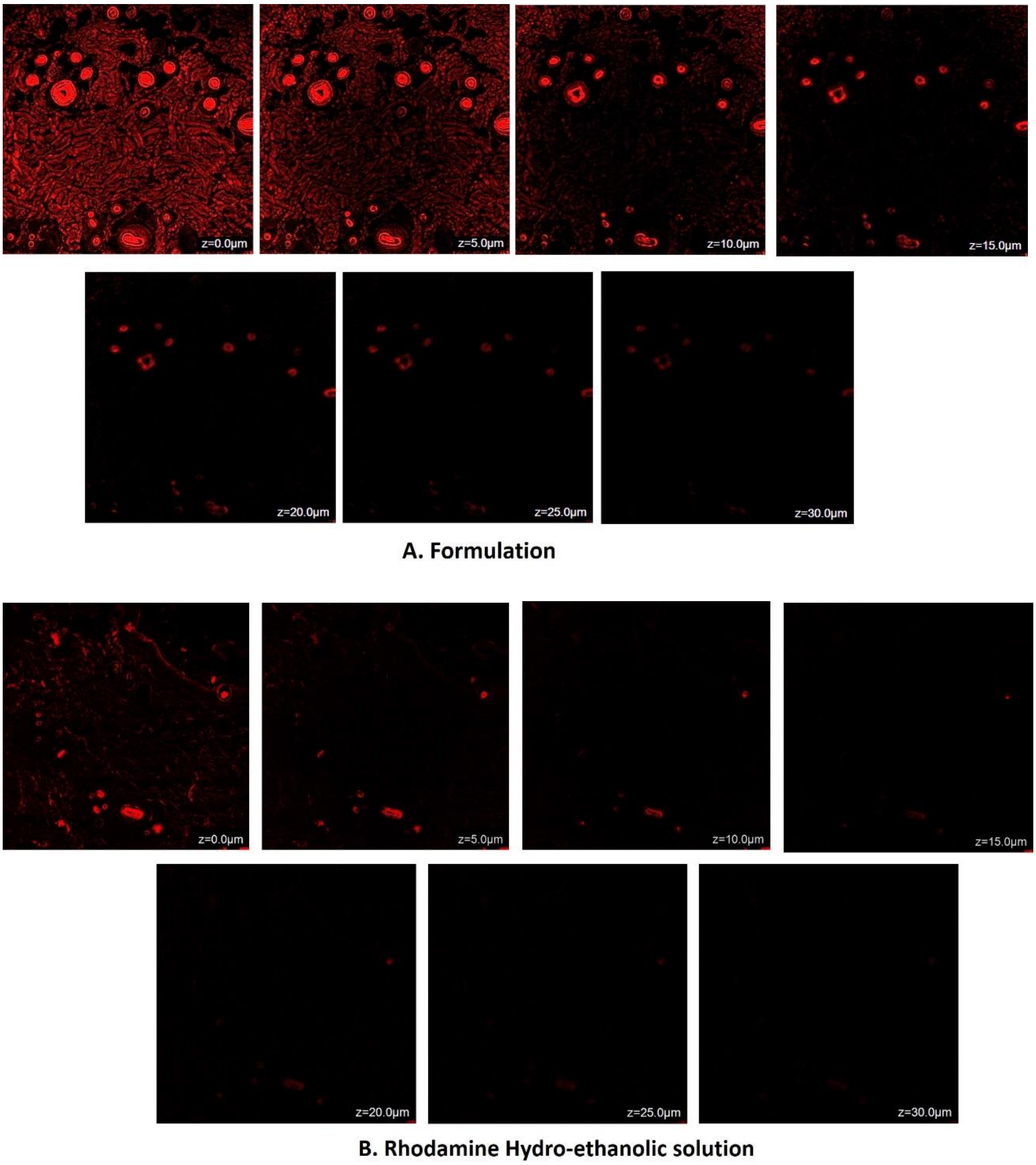

Figure S2. Confocal images of rats with skin surfaces that have been treated with (**A**) Rhodamine B-loaded optimized transfersomes gel formulation and (**B**) Rhodamine B hydroalcoholic solution.
